# Pharmacological and Non-pharmacological Treatment for Decompensated Heart Failure: What Is New?

**DOI:** 10.1007/s11897-017-0328-x

**Published:** 2017-04-18

**Authors:** Parin Shah, Pierpaolo Pellicori, Joseph Cuthbert, Andrew L. Clark

**Affiliations:** Department of Cardiology, Hull York Medical School, Hull and East Yorkshire Medical Research and Teaching Centre, Castle Hill Hospital, Cottingham, Kingston upon Hull, HU16 5JQ UK

**Keywords:** Acute heart failure, Pharmacological treatments, Non-pharmacological treatments, Dyspnoea at rest

## Abstract

**Purpose of the Review:**

Acute heart failure (AHF) is a life-threatening clinical condition that requires prompt medical attention. The aim of the current review is to summarise the results of recent clinical trials conducted in patients with AHF.

**Recent Findings:**

Several novel compounds have apparently beneficial acute effects on cardiovascular haemodynamics and patients’ symptoms, but their administration has not yet translated into improved survival and has been deleterious in some cases.

**Summary:**

The management of patients with AHF is challenging and reflects the heterogeneity of patient’s presentation, the complexity and severity of a multi-organ syndrome, and the limited therapeutic options, usually restricted to a combination of diuretics and vasodilators. Ongoing trials of novel treatments may provide evidence of an effect on outcomes.

## Introduction

Acute heart failure (AHF) is a life-threatening clinical condition that requires prompt medical attention [1•]. For patients with chronic heart failure due to left ventricular systolic dysfunction, there is a huge wealth of data demonstrating unequivocally that some interventions improve survival and symptoms. The cumulative effect of modern therapy has a dramatic effect on survival. By contrast, there is still no good evidence that any treatment for *acute* heart failure improves prognosis. How has this come about?

Patients presenting with AHF are heterogeneous. Many clinicians asked to describe a notional patient with AHF describe a patient with acute pulmonary oedema. However, pulmonary oedema is a less common presentation than might be thought: most patients presenting with acute heart failure do so because they have fluid retention (and peripheral oedema) as their dominant symptom; they are not short of breath at rest [2, 3].

Clinical trials in patients with AHF often aim to recruit patients who have resting breathlessness (which seems to suggest the intention to recruit patients with pulmonary oedema) [4••, 5••]. However, the trial entry criteria often then seek to exclude those with an obvious precipitant for their pulmonary oedema, such as acute ischaemia or an arrhythmia. This creates a problem: almost by definition, acute pulmonary oedema has a precipitant of some sort.

A related issue is that patients with acute pulmonary oedema often present out-of-hours, at a time when it is difficult to go through complex trial procedures and when research staff are often not at work. Such patients are often extremely unwell, and not well placed to give informed consent prior to trial entry.

The treatment for acute pulmonary oedema may not have changed much for 40 years, but nor has the natural history: with standard treatment (usually an intravenous loop diuretic and perhaps a nitro vasodilator), patients often rapidly recover [6]. A consequence for a clinical trialist interested in, say, relief of breathlessness as a primary endpoint is that by the time a patient is recruited, many hours have passed since the initial presentation, by which time the patient’s symptoms have largely settled.

The majority of patients presenting with AHF have fluid retention rather than pulmonary oedema (although may obviously have pulmonary congestion). In such patients, an intravenous diuretic given over many days is the standard treatment. There have been few clinical trials designed to test treatments in this clinical scenario.

The current European Society of Cardiology (ESC) guidelines for the management of acute heart failure stress that diagnosis and identification of precipitants should take place in parallel with treatment.^1^ However, most of the treatments for AHF are still opinion, rather than evidence, based. Several promising therapies have been shown to improve cardiovascular haemodynamics and patients’ symptoms, but the acute effects have not yet been shown to translate into improved survival; some have actually been deleterious [7••, 8]. The aim of the present review is to discuss some of the novel agents and interventions which might have a future role in the management of patients with AHF.

### Current Treatments

Intravenous loop diuretics are the cornerstone of treatment of congestion in patients with AHF. However, it is still not clear what the best treatment regime might be. In the DOSE trial, patients admitted with AHF with a blood pressure greater than 90 mmHg were randomised to a low (total intravenous furosemide dose equal to previous daily oral dose) or a high dose of loop diuretic (total daily intravenous furosemide dose 2.5 times previous daily oral dose), either given as a bolus or continuous infusion. There was no significant difference either for the safety endpoint of worsening renal function or the efficacy endpoint of global assessment of symptoms between these groups. However, patients receiving higher doses had a greater mean weight loss and mean fluid loss, at a price of some transient worsening in renal function [9•]. The current ESC-HF guidelines recommend the smallest dose of diuretic required to provide clinical effect [1•].

Vasodilators are the second most commonly used treatment in patients with AHF, especially in the hypertensive patient, and particularly in those patients with pulmonary oedema. Registries suggest that they are used in only around one out of five patients with AHF [1•, 10]. Nearly 20 years ago, Cotter and colleagues randomised patients with pulmonary oedema to high-dose isosorbide dinitrate (3 mg bolus administered intravenously every 5 min) or high-dose furosemide (80 mg bolus administered intravenously every 15 min) plus isosorbide dinitrate (dose of 1 mg/h). Compared with low-dose nitrates, high-dose nitrates decreased myocardial infarction (MI) and the need for mechanical ventilation without any effect on mortality. Higher dose of nitrates was also associated with a lower heart rate (HR), higher oxygen saturation, and lower respiratory rate [11•].

Some patients with AHF may present with a low blood pressure or in shock. In these scenarios, a prompt identification of precipitating cause (for instance, an acute myocardial infarction, or pulmonary embolism) is paramount. For those cases in whom cardiac output is severely reduced and organ perfusion is compromised, vasopressors, like noradrenaline, and inotropes, like levosimendan or dobutamine, can be used with caution. However, such evidence as there is from randomised trials suggests that the use of positive inotropic agents is associated with a worse prognosis [12]. In selected cases that do not stabilise despite inotropic support, mechanical circulatory support or urgent heart transplant may be considered [1•].

### Recent Advances in Heart Failure Treatment

Given the high mortality and economic burden of patients admitted with AHF, and the lack of effective drugs, other than those mentioned above, there is an urgent need for novel therapies and better clinical trial designs [4••]. When designing clinical trials for novel therapies in AHF, several factors need to be considered. As well as patient heterogeneity, “standard” therapy for AHF is not standardised, leading to potentially very variable treatment of patient in the control arms of studies. Clinical trials often use short-term primary endpoints, such as changes in symptoms, which are susceptible to patient or physician subjective bias [13]. On the other hand, it is difficult to picture how the single administration of a drug given at presentation might affect more robust (and perhaps clinically important) endpoints, such as early heart failure readmission and short-term (30–180 days) survival [5••].

## Pharmacological Therapies

### Natriuretic Peptides

#### Nesiritide

**Fig. 1:**
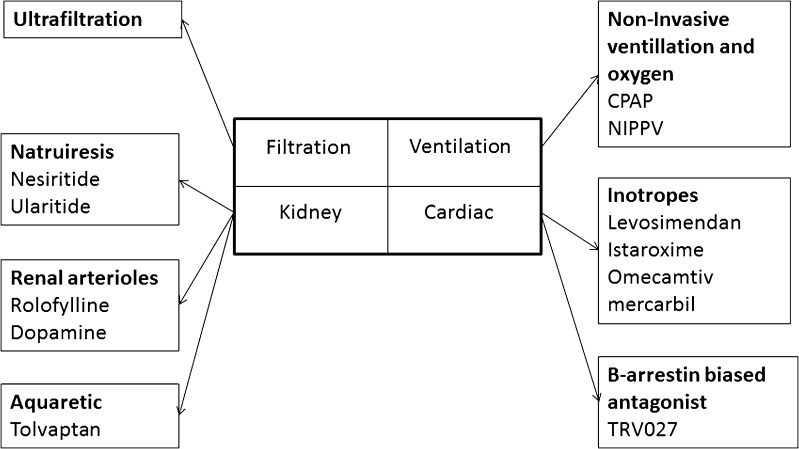
Novel therapies in acute decompensated heart failure. *CPAP* continuous positive airway pressure, *NIPPV* non-invasive positive pressure ventilation

Nesiritide is recombinant human brain natriuretic peptide (BNP). It has vasodilatory effects on the arteries and veins, enhances sodium excretion, and supresses both the renin-angiotensin-aldosterone and sympathetic nervous systems [14] (Fig. 1).

The VMAC study was very influential in getting nesiritide licenced for use in the USA. In VMAC, 432 patients with AHF were randomised to receive nesiritide, nitrate, or placebo. Nesiritide reduced pulmonary capillary wedge pressure and improved both dyspnoea and global status as assessed by a physician [15] (Table 1). However, nesiritide did not improve symptoms or mortality when compared to standard vasoactive medications [15]. In large part, because European authorities were reluctant to accept haemodynamic data as being sufficiently definitive to allow licencing, a large (>7000 patients) study, ASCEND-HF, was conducted in patients presenting with pulmonary congestion. Compared to placebo, nesiritide did not improve 30-day all-cause mortality, rehospitalisation for heart failure, or renal function. However, patients treated with nesiritide were more likely to have episodes of hypotension compared to placebo (26.6% nesiritide vs. 15.3% placebo, *p* < 0.001) [16]. Current guidelines do not endorse neseritide as a treatment of AHF [1•] (Table 1).

**Table 1:** Novel treatments in acute decompensated heart failure

Study	Pts	HFrEF (%)	Signs/symptoms	Other inclusion criteria	Placebo/comparator	Haemodynamic	Symptoms	Readm	No. of days alive and out of hospital	Mortality	Safety
Nesiritide											
VMAC 2002	498	85	NYHA = III to IV Signs of congestion^a^		Placebo	Improve	SOB: improved GCS: no change				
					Nitroglycerine	Improve	SOB: no change GCS: no change	30 days: no change		6 months: no change	Hypotension: no change Renal function: no change
ASCEND-HF 2011	7141	80	SOBAR, RR >20, pulmonary congestion		Placebo		SOB: no change	30 days: no change		30 days: no change	Hypotension: increased Renal function: no change
Ularitide											
SIRIUS I 2005	24		NYHA class III–IV		Placebo	Improve	SOB: improved				
SURIUS II 2006	221	>95	SOBAR	CI <2.5 l/min PCWP ≥18 mmHg	Placebo	Improve	SOB: improved				Hypotension: increased Renal function: no change
Levosimendan											
LIDO 2002	203	100		CI <1.9 l/min PCWP >24 mmHg	Dobutamine	Improve	SOB: no change		Greater	30 days: improved	
SURVIVE 2007	1327	100	Oliguria SOBAR	CI ≤ 2.2 l/min PCWP >18mmHG Inotropes	Dobutamine		SOB: no change		No change	31 and 180 days: no change	Hypotension: increased
REVIVE 2013	700	100	SOBAR despite diuretics		Placebo		SOB: improved		No change		Hypotension: increased
Istaroxime											
HORIZON-HF 2009	120	100	SBP: 90–150 mmHg		Placebo	Mixed results					
Seralaxin											
Pre-RELAX-AHF 2009	234	44–68	SOBAR, pulmonary congestion	SBP ≥125 mmHg Impaired renal function	Placebo		SOB: improved Weight loss: greater	No change		180 days: no change	
RELAX-AHF 2013	1161	55	SOBAR, pulmonary congestion	SBP ≥125 mmHg Impaired renal function	Placebo		SOB: improved	60 days: no change		180 days: no change	Hypotension: increased
TVR027											
Soergel et al. 2013	32		Stable HF SBP ≥ 100 mmHg	PCWP: ≥20 mmHg	Placebo	Improve					
Omecamtiv mecarbil											
ATOMIC HF 2016	606	100	SOBAR		Placebo		SOB: no change	No change		30 days: no change	
Rolofylline											
PROTECT pilot 2008	301		SOBAR, signs of congestion^a^	Impaired renal function	Placebo		SOB: greater Weight loss: greater	60 days: reduced		60 days: reduced	Renal function: no change
PROTECT 2010	2033	100	SOBAR	Impaired renal function	Placebo		SOB: no change	60 days: no change		60 days: no change	Increased seizures
Dopamine											
DAD-HF 2010	60	100	SOBAR NYHA class 4		Loop diuretic			60 days: no change		60 days: no change	Renal function: decreased
ROSE-HF 2013	360		1 symptom and sign of congestion^a^	Impaired renal	Placebo		SOB: no change	60 days: no change		60 days: no change	Renal function: no change
Tolvaptan											
EVEREST trial 2007	4133	100	Signs of volume expansion NYHA class III/IV				SOB: improved Weight: reduced	No change		No change	Renal function: decreased
TACTIC HF 2016	257	Any LVEF	SOBAR, signs of congestion^a^	Raised BNP			SOB: no change				Renal function: decreased
Non-invasive ventilation											
Gray et al. 2008	1069		Cardiogenic shock and pulmonary oedema		Oxygen		SOB: greater reduction			7 and 30 days: no change	
Ultrafiltration											
UNLOAD 2007	200	70	Hypervolemia, signs of congestion^a^		Diuretic		SOB: no change Weight loss: greater loss	Reduced			Renal function: no change
CARRESS-HF 2012	188	Any LVEF	ADHF signs of congestion	Renal impairment	Diuretic		SOB: no change Weight loss: no change				Renal function: decreased
AVOID-HF 2015	224	N/A	ADHF, signs of congestion^a^		Diuretic		Weight loss: no change	No change	30 and 90 days: no change	90 days: no change	Renal function: no change

*Pts* patients, *HFrEF* Heart failure with reduced ejection fraction, *Readm* readmission *NYHA* New York Heart Association, *SOB* shortness of breath, *GCS* global clinical score, *SOBAR* shortness of breath at rest, *CI* cardiac index, *PCWP* pulmonary capillary wedge pressure, *ADHF* acute decompensated heart failure, *CXR* chest X-ray, *LVEF* left ventricular ejection fraction, *N/A* not applicable

^a^Signs of congestion: pitting oedema >2=, jugular venous distension >8 cm, pulmonary oedema or pleural effusion on chest X-ray, paroxysmal nocturnal dyspnoea, orthopnoea, respiratory rate >20

#### Ularitide

Another endogenous natriuretic peptide, urodilatin, is produced in the kidneys. It binds to natriuretic peptide type A receptors in the inner medullary collecting duct. It increases intracellular cyclic guanosine monophosphate (cGMP) which inhibits sodium reabsorption via an amiloride-sensitive channel. Ularitide is a synthetic version of urodilatin [17] (Fig. 1).

The phase IIa randomised double-blind SIRIUS I study enrolled 24 patients; compared to placebo, both 15- and 30-ng/kg/min doses of ularitide decreased pulmonary capillary wedge pressure (PCWP), right atrial pressure (RAP), and N-terminal pro BNP from baseline [18] (Table 1).

A subsequent phase IIb (SIRIUS II) randomised, double-blind, placebo-controlled trial in 221 patients with AHF who had low cardiac index (<2.5 l/min) and raised PCWP (≥18 mmHg) found that ularitide was associated with a rapid and significant decrease in PWCP and, at higher doses, systemic vascular resistance (SVR) [19] (Table 1). There was no effect of ularitide on renal function during the 24-h infusion and in the 48 h of follow-up, but some patients in the 30 ng/kg/min infusion group (*N* = 7, 13%) had their drug temporarily interrupted due to hypotension [20]. Whether ularitide improves clinical status and 180-day survival in patients with decompensated heart failure is currently under evaluation in the TRUE AHF (NCT01661634).

### Inotropic Agents

#### Levosimendan

Levosimendan sensitises troponin C to calcium thereby improving myocardial contractility and haemodynamics. Levosimendan also has vasodilatory and anti-ischaemic properties [21] (Fig. 1).

Early data from LIDO suggested that compared to dobutamine, levosimendan improved cardiac haemodynamics and 30- and 180-day mortality [21] (Table 1). The effect on mortality was not confirmed in the larger SURVIVE trial (*N* = 1327 patients) [22]. More recently, in the REVIVE study, levosimendan was compared to placebo in 700 patients with AHF with a mean LVEF of 23%, who remained dyspnoeic at rest despite treatment with intravenous diuretics and in some cases vasodilators (13%) or inotropes (11%). Compared to placebo, levosimendan had a greater symptomatic improvement, but led to a higher incidence of hypotension, higher heart rate, and subsequent risk of developing atrial and ventricular arrhythmias, thus increasing the risk of death [23].

The current ESC guidelines on heart failure recommend levosimendan in cardiogenic shock in combination with other vasopressors and over dobutamine only if reversal of beta-blockers is need to improve the hypoperfusion [1•].

#### Istaroxime

Istaroxime is a novel intravenous agent which both inhibits sodium/potassium ATPase (much as cardiac glycosides) and stimulates sarcoplasmic reticulum calcium adenosine triphosphatase isoform 2a (SERCA2a), thereby stimulating the re-uptake of calcium into the sarcoplasmic reticulum during diastole. The combined mechanism of action means that istaroxime has both inotropic and lusitropic properties [24] (Fig. 1).

A phase II, randomised, controlled trial (HORIZON-HF) evaluated the short-term effects of istaroxime in 120 patients with AHF and a left ventricular ejection fraction ≤35%. Compared to placebo, all doses of istaroxime (0.5, 1.0, and 1.5 μg/kg/min) caused a fall in PCWP and heart rate and increased systolic blood pressure. Although istaroxime reduced left ventricular volumes, and indices of diastolic function on echocardiography from baseline, compared to placebo, there was no significant change in these variables [24] (Table 1). Vomiting and pain at the infusion site were the most common side-effect reported. A study aiming to recruit 120 patients with AHF with LVEF less than 40%, who are admitted with dyspnoea at rest or minimal exertion needing intravenous diuretics, is currently planned. The primary endpoint is change of diastolic function (E/Ea ratio assessed by tissue Doppler on echocardiography) (NCT02617446).

### Other Novel Pharmacological Agents

#### Serelaxin

Serelaxin is a recombinant form of the naturally occurring relaxin-2 involved with the adaptation of cardiovascular and renal function during pregnancy [25] (Fig. 1).

In the Pre-RELAX-AHF study, 230 patients with AHF (admitted with dyspnoea at rest or on minimal exertion, evidence of pulmonary congestion on chest radiography, raised natriuretic peptides, and a systolic blood pressure >125 mmHg) were randomised to standard care followed by 48-h intravenous infusion of placebo (*n* = 62) or different doses of serelaxin (10, 30, 100, or 250 μg/kg per day). Compared to placebo, patients randomised to 30-μg/kg/day dose had the greatest improvement in shortness of breath and resolution of signs of congestion (physician-assessed jugular venous pressure, rales, and oedema), reduction in body weight, and use of intravenous diuretics [25]. A subsequent phase III trial (RELAX-AHF) of 1161 patients with AHF, recruited within 16 h of admission with dyspnoea at rest, pulmonary congestion of chest X-ray raised plasma levels of natriuretic peptides, and mild to moderate impaired renal dysfunction, compared the infusion of 30 μg/kg/day for 48 h to placebo. Serelaxin improved breathlessness slightly more than placebo, although it was difficult to understand the clinical significance. In a post hoc analysis, there was a 37% reduction in all-cause mortality at 180 days [26]. In a sub-analysis of the trial, Metra and colleagues also reported a significant reduction in NTproBNP, high-sensitivity troponin T, and incidence of worsening renal function (serum creatinine increase of 0.3 mg/ or a cystatin C increase of 0.3 mg/l) at day 2 in those randomised to serelaxin [27]. The results are by no means definitive. Several patients were lost to follow-up and the mortality effect was of borderline statistical significance (Table 1). A large, multicentre, phase III trial (RELAX-AHF-2) is currently ongoing and plans to recruit more than 6000 patients. The primary endpoints are cardiovascular death first occurrence of worsening heart failure.

#### TVR027

The angiotensin II type 1 receptor (AT1R) is a G protein-coupled receptor that mediates the biological effects of angiotensin II. There are two pathways down-stream from the receptor: firstly, signalling mediated via Gq protein causes vasoconstriction and cardiac hypertrophy. and secondly, signalling mediated via β-arrestin-2 causes modest inotropic and cardioprotective effects (stimulation of cardiomyocyte proliferation, activation of the pro survival kinase, Akt, and reduction in apoptosis). Angiotensin receptor blockers block both signalling pathways: Gq, causing reduction in vascular tone, and β-arrestin-2, causing reduction in cardiac contractility and cardio protective effects. TRV027 is a novel β-arrestin-biased antagonist of the AT1R, meaning that it antagonises the Gq protein pathway whilst stimulating β-arrestin-2 [28] (Fig. 1).

In a study by Soergel and colleagues in patients with chronic heart failure, compared to placebo, TVR027 caused a dose-dependent decrease in PCWP with no change in cardiac index (CI) and a reduction in mean arterial pressure [29] (Table 1). The BLAST-AHF is a phase II study in which approximately 500 patients with AHF and systolic blood pressure ≥120 mmHg will be randomised to different doses of intravenous TRV027 (1, 5, or 25 mg/h) or placebo for at least 48 h, and up to 96 h, to evaluate its efficacy on symptoms and plasma levels of natriuretic hormones [28].

#### Omecamtiv Mecarbil

Omecamtiv mecarbil is a selective cardiac myosin activator which increases myocardial contractility without increasing myocardial oxygen demand [30] (Fig. 1). In the ATOMIC-AHF study, conducted in 606 patients with AHF, LVEF <40%, and raised natriuretic peptides, the use of omecamtiv mecarbil did not have influence symptoms or mortality compared to placebo [30]. Although it increased left ventricular systolic ejection time and reduced end-systolic LV dimension, those randomised to omecamtiv mecarbil had higher troponin levels at 48 h [30]. Nevertheless, side-effects were the same as placebo (Table 1). The results of COSMIC-HF study which compared omecamtiv mecarbil to placebo in 544 patients with heart failure with reduced ejection fraction and raised BNP are awaited (NCT01786512).

#### Rolofylline

Patients with AHF with worsening renal function have a poor prognosis. Adenosine acts on the adenosine A1 receptors in the renal arterioles to reduce renal blood flow thereby reducing glomerular filtration rate and increasing renin production. Stimulation of the adenosine A1 receptors also increases sodium reabsorption in proximal tubule. Antagonising the adenosine A1 receptor by rolofylline might thus improve renal blood flow, perhaps improving renal function and diuresis [31] (Fig. 1).

In the PROTECT pilot study, different doses rolofylline or placebo were administered daily for up to 3 days in patients with AHF; those who received the 30-mg dose of rolofylline had a greater relief of dyspnoea and less worsening of renal function, compared to placebo [31].

These results were not confirmed in a subsequent phase III trial enrolling >2000 patients, in which 30 mg of rolofylline or placebo was administered as a 4-h intravenous infusion daily for (up to 3 days). Seizures being more common in rolofylline group [32] (Table 1). There will, unfortunately, continue to be no direct treatment for renal dysfunction in patients with AHF.

#### Dopamine

Worsening renal failure is common in patients with AHF. Dopamine is an endogenous catecholamine and, at low doses, increases glomerular filtration rate by vasodilation of renal blood vessels [33, 34] (Fig. 1).

In the DAD-HF study, 60 patients with a mean LVEF of 35%, admitted with recent onset of dyspnoea (<6 h) with other signs of congestion, were randomised to either high-dose furosemide or low-dose furosemide combined with low-dose dopamine. The combination of low-dose furosemide and low-dose dopamine had significantly lower incidence of worsening renal function (>0.3-mg/dl rise in serum creatinine from baseline to 24 h). The mean hourly urine output and the dyspnoea scores were similar in both groups but there was no difference in 60-day mortality and rehospitalisation [35]. To answer the question of whether low-dose furosemide or dopamine reduce the incidence of worsening renal function, the DAD-HF II study of 161 patients included patients on low-dose furosemide on its own. High-dose furosemide had an increased incidence of worsening renal function compared to low-dose furosemide, with or without dopamine [36] (Table 1).

In the ROSE AHF study, 360 patients admitted with AHF regardless of LVEF who had at least one symptom (dyspnoea, orthopnoea, or oedema) and one sign (rales, oedema, ascites, or pulmonary vascular congestion on chest radiography) of acute heart failure, renal dysfunction (glomerular filtration rate of 15–60 ml/min/1.73 m^2^), and on background diuretic therapy were randomised to dopamine, nesiritide, or placebo. Compared to placebo, dopamine did not affect the 72-h cumulative urine volume, change in cystatin C level at 72-h or 60-day mortality, and readmission with heart failure [34] (Table 1).

#### Tolvaptan

In many patients with AHF, loop diuretics cause hyponatraemia, a poor prognostic sign, and a feature suggesting that diuretic therapy is likely to fail. An agent that enhances water excretion whilst having no effect on sodium excretion (an aquaretic) might thus be beneficial. Tolvaptan is an oral vasopressin-2 receptor antagonist which inhibits anti diuretic hormone leading to excretion of free water [37] (Fig. 1).

However, in the large EVEREST trial (*N* = 4133 patients), compared to placebo, tolvaptan did not reduce the primary endpoints of all-cause mortality and combined cardiovascular death and rehospitalisation due to heart failure, although it improved symptoms and serum sodium levels, without increasing adverse events [37]. In TACTICS-HF, 257 patients with AHF, regardless of LVEF admitted with dyspnoea at rest and one other sign or symptom of congestion (orthopnoea, oedema, elevated jugular venous pulse, rales, or congestion on chest radiograph), were randomised to receive three doses of tolvaptan over 48 h in addition to standardised background furosemide therapy. Whilst tolvaptan caused greater weight loss (tolvaptan −3.7 (4.4) kg vs placebo −2.5 (3.2) kg, *p* = 0.07), it did not affect fluid loss (tolvaptan 1757 (1670) ml vs placebo 1401 (1387), *p* = 0.11)) or symptoms, and was associated with a higher chance of worsening renal function [38] (Table 1).

The current ESC guidelines on HF advise considering tolvaptan in AHF in patients with volume overload and resistant hyponatraemia [1•]. The vasopressin antagonists have not yet found a clearly defined role in managing patients with heart failure. It is possible that they may be particularly helpful in patients with marked hyponatraemia, but the right trial has not yet been conducted.

## Non-pharmacological Therapies

### Ventilatory Support: Oxygen and Non-invasive Ventilation

Oxygen has been used liberally pre hospital and in the emergency departments in the perception that it relieves dyspnoea and improves myocardial oxygenation despite normal oxygen saturation. However, supplemental oxygen and assisted ventilation should be reserved for patients with hypoxaemia. In the very few studies that have systematically examined the effects of increasing Fio_2_, oxygen supplementation causes a fall in cardiac output and increases in SVR and cardiac filling pressures [39] (Fig. 1).

In a study by Gray and colleagues, 1069 patients with AHF admitted with cardiogenic pulmonary oedema and arterial pH of less than 7.35 were randomised to standard oxygen therapy, continuous positive airway pressure (CPAP), or non-invasive intermittent positive pressure ventilation (NIPPV). Compared to standard oxygen therapy, non-invasive ventilation (both CPAP and NIPPV) did not alter 7- and 30-day mortality, but did lead to greater reduction in dyspnoea, heart rate, and hypercapnia [40] (Table 1). A subsequent meta-analyses on the use of non-invasive positive pressure ventilation (NIPPV) showed a reduction in hospital mortality compared to standard treatment [41, 42].

### Ultrafiltration

Ultrafiltration (UF) can be used to remove the excess salt and fluid of patients with fluid retention, even if they are resistant to high doses of diuretics. In the UNLOAD trial, which tested the safety and efficacy of veno-venous ultrafiltration versus standard diuretic therapy in 200 congested patients with AHF, ultrafiltration had a more pronounced effect on weight reduction and fluid loss than standard therapy, and was associated with a decrease in 90-day rehospitalisation for HF [43] (Fig. 1).

In a subsequent study, CARRESS-HF, conducted in 188 patients with AHF with signs of congestion (at least two criteria: 2+ peripheral oedema, or more; jugular venous pressure greater than 10 cm of water; or pulmonary oedema or pleural effusion on chest radiography) and worsening renal function (increase in the serum creatinine level of at least 0.3 mg per decilitre) compared to standard pharmacological therapy, UF led to a worsening in renal function with no significant difference in weight loss between the two groups [44]. There was a higher number of adverse events in the ultrafiltration group, mainly due to increased incidences of kidney failure, bleeding, and catheter complications, but the deterioration in renal function could simply be due to serum concentration (Table 1).

Recently, the AVOID-HF trial was terminated early by the sponsor when 224 of the 800 planned patients with AHF had been enrolled. Patients had to have at least two signs of congestion (pitting oedema ≥2+ of the lower extremities, jugular venous distension >8 cm, pulmonary oedema or pleural effusion on chest X-ray, paroxysmal nocturnal dyspnoea, more than two pillow orthopnoea, and respiratory rate more than 20 breathes per minute). The preliminary data showed no advantage to UF over adjustable diuretic treatment; also, more patients in the UF arm experienced adverse events [45] (Table 1).

The current ESC guidelines suggest ultrafiltration to be considered on patients who fail diuretic therapy [1•]. It remains a therapy that appears promising, but has yet to find a definitive role. It is possible that it may have a niche application in managing patients with very severe and unresponsive fluid retention, but it is unlikely ever to achieve widespread use as standard therapy.

### Conclusion

The management of patients with AHF is challenging and reflects the heterogeneity of patient’s presentation, the complexity and severity of a multi-organ syndrome, and the limited therapeutic options, usually restricted to a combination of diuretics and vasodilators. Recent trials of novel pharmacological and non-pharmacological therapies have shown a number of possible agents that might offer beneficial haemodynamic responses; however, it is not at all clear that beneficial haemodynamic responses translate into improved clinical outcomes, whether in terms of short-term symptom relief or longer-term outcome. More trials are needed that will have to meet a number of criteria. The patients included in a trial must be carefully phenotyped so we know exactly to whom particular sets of trial results refer; they must be powered for relevant clinical endpoints (such as robust markers of symptoms severity, or length of stay); and they must answer robustly framed questions about outcome (such as days alive and out of hospital at 6 months).
